# Efficient Synthesis with Green Chemistry Approach of Novel Pharmacophores of Imidazole-Based Hybrids for Tumor Treatment: Mechanistic Insights from In Situ to In Silico

**DOI:** 10.3390/cancers14205079

**Published:** 2022-10-17

**Authors:** Majid Khan, Syed Raza Shah, Faizullah Khan, Sobia Ahsan Halim, Shaikh Mizanoor Rahman, Mohammad Khalid, Ajmal Khan, Ahmed Al-Harrasi

**Affiliations:** 1 Natural and Medical Sciences Research Center, University of Nizwa, 616 Birkat Al Mauz, Nizwa P.O. Box 33, Oman; 2 H.E.J. Research Institute of Chemistry, International Center for Chemical and Biological Sciences, University of Karachi, Karachi 75270, Pakistan; 3 Department of Pharmacy, Abdul Wali Khan University Mardan, Mardan 23200, Pakistan; 4 Department of Pharmaceutics, College of Pharmacy, King Khalid University, Abha 62529, Saudi Arabia

**Keywords:** imidazole-based hybrids with hydroxyethyl and pyrimidine, anticancer, mechanistic studies, cytotoxicity, in silico molecular docking

## Abstract

**Simple Summary:**

Here, we report the eco-friendly synthesis and antitumor potential of the imidazole hybrids of pyrimidine. The results showed that all the compounds possess excellent inhibition of tumors, promoting enzymes hCA-IX and hCA-II. Furthermore, the selectivity index showed that compounds **7**, **10**, and **11** are selective inhibitors of hCA-IX, while compound **2** is a selective inhibitor of hCA-IX. More importantly, all the active inhibitors are toxic to the breast cancer cell line and non-cytotoxic for the normal breast cell line. These compounds would be a suitable choice to investigate in the in vivo models to check their efficacy against these particular targets. These newly identified human carbonic anhydrase inhibitors have potential to be considered as therapeutic leads for the treatment of CA-related diseases, especially for breast and lung tumors and glaucoma. Furthermore, lead optimization and preclinical and clinical investigations of these compounds are necessary to develop potential drug entities for the treatment of cancer.

**Abstract:**

Imidazole-based pyrimidine hybrids are considered a remarkable class of compounds in pharmaceutical chemistry. Here, we report the anticancer bioactivities of eleven imidazole-based pyrimidine hybrids (**1**–**11**) that specifically target cytosolic carbonic anhydrase (CAs) isoenzymes, including human CA-II and human CA-IX (hCA-II, and hCA-IX). A highly eco-friendly aqueous approach was used for the formation of a carbon–carbon bond by reacting aromatic nitro group substitution of nitroimidazoles with carbon nucleophiles. The in vitro results indicate that this new class of compounds (**1**–**11**) includes significant inhibitors of hCA IX with IC_50_ values in the range of 9.6 ± 0.2–32.2 ± 1.0 µM, while hCA II showed IC_50_ values in range of 11.6 ± 0.2–31.1 ± 1.3 µM. Compound **2** (IC_50_ = 12.3 ± 0.1 µM) showed selective inhibition for hCA-II while **7**, **8**, and **10** (IC_50_ = 9.6–32.2 µM) were selective for hCA-IX. The mechanism of action was investigated through in vitro kinetics studies that revealed that compounds **7**, **3**, **11**, **10**, **4**, and **9** for CA-IX and **1**, **2**, and **11** for CA-II are competitive inhibitors with dissociation constant (Ki) in the range of 7.32–17.02 µM. Furthermore, the in situ cytotoxicity of these compounds was investigated in the human breast cancer cell line MDA-MB-231 and compared with the normal human breast cell line, MCF-10A. Compound **5** showed excellent anticancer/cytotoxic activity in MDA-MB-231 with no toxicity to the normal breast cells. In addition, in silico molecular docking was employed to predict the binding mechanism of active compounds with their targets. This in silico observation aligned with our experimental results. Our findings signify that imidazole-based hybrids could be a useful choice to design anticancer agents for breast and lung tumors, or antiglaucoma compounds, by specific inhibition of carbonic anhydrases.

## 1. Introduction

Human carbonic anhydrases (hCAs: EC 4.2.1.1) are zinc-containing enzymes that catalyze the reversible hydration of carbon dioxide to bicarbonate and protons. Zinc plays an essential part of the reaction by acting as a Lewis acid to ionize the water molecule. To date, fourteen diverse isoforms (CA-I, CA-II, CA-III, CA-VII, CA-IX, and CA-XIII) of CAs have been identified with diverse molecular features (quaternary structure), organ and tissue distribution, subcellular localization, catalytic properties, and the ability to interact with different classes of inhibitors [1,2]. CAs are present in prokaryotes and eukaryotes and are classified into seven unrelated gene families. So far, eight families has been discovered, which are named α-CAs to ι-CAs [3,4].

Mammalian CAs regulate various physiological functions including pH control, bicarbonate metabolism, and cellular pressure [5]. The abnormal or upregulated expression of CAs has a negative effect which often leads to chronic diseases, such as glaucoma, edema, neurological disorders, and epilepsy [6,7]. Recently, the role of CA-IX was studied in the growth of solid tumors in response to hypoxia-inducible factor-1α [8,9] This hypoxic condition triggers the overexpression of CA isozymes, i.e., IX, which amplifies the progression of cancer cells [10]. CA-IX also plays a key role in the tumor growth of epithelial cells from breast (MDA-MB-231) and lung (A549) cancers by acidifying the tumor microenvironment [11]. Similarly, the role of CA –XII is well-established for its anti-glaucoma properties [12,13]; dorzolamide is a widely used hCA-II inhibitor of glaucoma treatment [13]. Hence, the inhibition of CAs is a fascinating approach to discover new anticancer and anti-glaucoma agents to eradicate cancer or to lower the rate of cancer progression and other life-threatening diseases [14,15,16].

**Figure 1:**
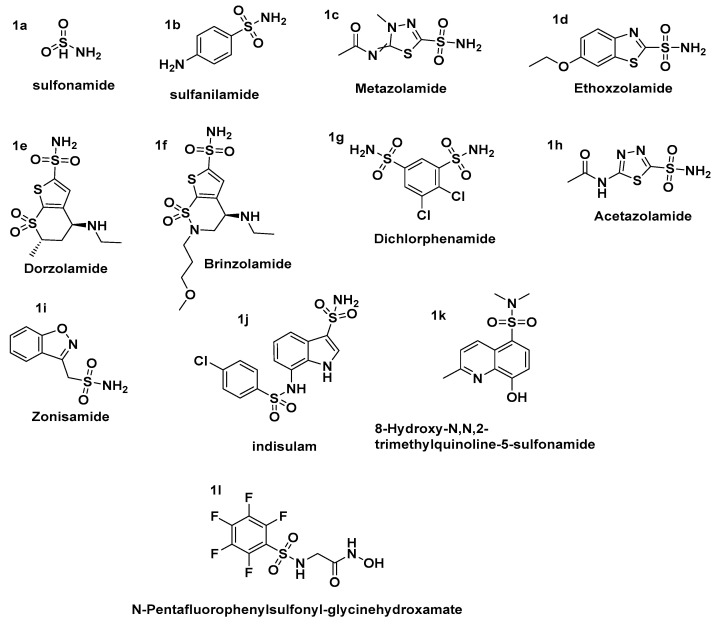
Some of the known sulfonamide derivatives as potent carbonic anhydrase inhibitors.

Known α-CA inhibitors include various anions, phenol, hydroxyurea, carboxylates, organic phosphates, and phosphonates [17,18,19]. The first discovered organic CA inhibitors were sulfonamide (Figure 1a), sulfanilamide (Figure 1b), [20], and variations on the sulfonamide structure (Figure 1c–j). By substituting other drug moieties, several other CA inhibitors were designed, such as hydroxysulfonamides (Figure 1K) and hydroxamates (Figure 1l) [18,21,22,23]. Those sulfonamide-, hydroxysulfonamide-, and hydroxamate-bearing therapeutic CA inhibitors mainly coordinate with the zinc ion present in the active site of CAs [24]. The treatment of glaucoma requires a high dose of sulfonamide drugs to effectively reduce intraocular pressure. Those drugs have shown poor specificity for the targeted isozyme, therefore causing a wide range of undesirable side effects, including altered taste, malaise, fatigue, depression, and anorexia [25,26]. In addition, some of the marketed sulfonamide drugs cause allergies in a significant number of people [27,28]. Thus, the discovery of a new class of non-sulfonamide CA inhibitors, potentially with more controlled specificity for different CA isozymes, could lead to the development of useful alternatives to existing drugs. Therefore, with the aim to design a novel class of CA inhibitors with structural diversity, the imidazole moiety was hybridized with hydroxyethyl and pyrimidine because imidazole possesses biological and pharmaceutical importance, and its nucleus forms the main structure of several well-known components in human body, including histidine, vitamin B12, DNA bases, purines, histamine, and biotin. It also constitutes the structure of many natural/synthetic drugs, i.e., cimetidine, azomycin, and metronidazole. Imidazole-containing drugs have a broader scope in remedying various dispositions in clinical medicine [26]. Similarly, pyrimidine is also a heterocyclic compound that has shown diverse biological activities, such as antimicrobial, CNS-depressant, anti-inflammatory, analgesic, anti-convulsant, anticancer, anti-helmentic, antioxidant, and herbicidal [27]. The pyrazole-based imidazole derivatives are reported as potent inhibitors of hCA-IX and hCA-II [29]. Similarly, Shao, et al. reported pyrimidine–benzimidazol hybrids as potent anticancer agents by evaluating the derivatives against human cancer cell lines including MCF-7, MGC-803, EC-9706, and SMMC-7721 [30]. Due to the prominent role of the imidazole ring in anticancer agents, we have evaluated the designed imidazole-based pyrimidine hybrids for their anticancer potential through in vitro inhibition of human CAs, followed by kinetic studies. Moreover, their cytotoxic behavior was studied in situ in the human breast cancer cell line (MDA-MB-231) and compared with the human normal breast cell line (MCF-10A). Subsequently, their targeting mechanism was explored through structural bioinformatics methods. This new class of CA inhibitors needs further investigation to develop a non-sulfonamide-based therapeutic inhibitor of CAs.

## 2. Materials and Methods

### 2.1. Chemistry: General Procedure for the Synthesis of Compounds

All the reagents and compounds used in the synthesis were received directly from the suppliers and used without further purification. Methanol, pure ethanol, and other solvents were also acquired in sufficient purity from various sources and employed in the reaction medium without purification. All the targeted compounds were resynthesized in Anton Paar Microwave Synthesis Reactor. The melting points were taken on Stuart melting point apparatus SMP10. IR spectra (KBr) were recorded on a Bruker FT-IR IFS48 spectrophotometer. EI mass spectra were attained using HR-ESIMS: Agilent Technologies, 6530. Exact mass and data are presented as *m/z*. ^1^H-NMR and ^13^C-NMR spectra were recorded in DMSO-d_6_ by using Bruker 600 MHz and 150 MHz spectrophotometers, respectively. Splitting patterns are presented as follows; s, singlet; d, doublet; dd, double doublets; t, triplet; and m, multiplet. Chemical shifts are described in δ (ppm) and coupling constants are calculated in Hz. The progress in all the reactions was observed via TLC, which was performed on 2.0 × 5.0 cm aluminum sheets pre-coated with silica gel 60F_254_ to a thickness of 0.25 mm (Merck). The chromatograms were visualized by UV light (254–366 nm) or iodine vapors. The aforementioned compounds were successfully synthesized and characterized.

**Scheme 1:**
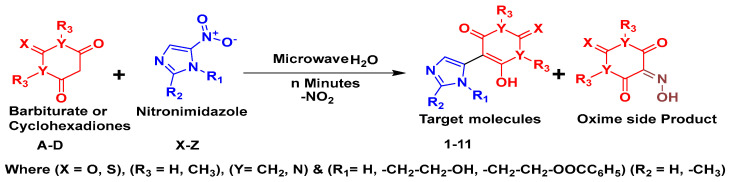
The water-based green reaction under microwave condition.

**Figure 2:**
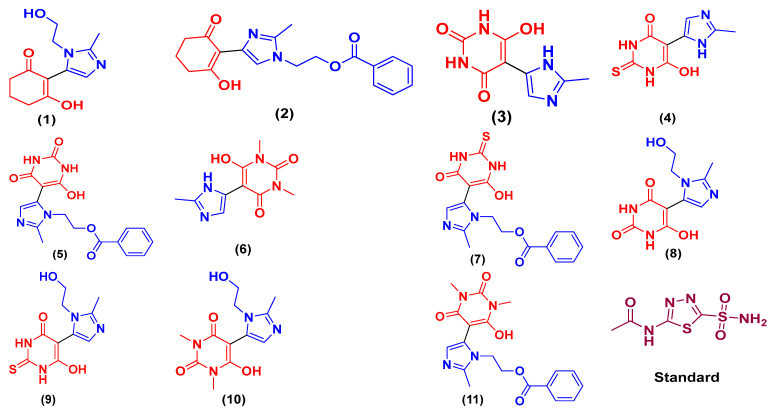
The 2D structures of the synthesized imidazole hybrids are presented.

A G10 vial of the Anton-Paar microwave synthesizer containing 6 mL of distilled water was charged with 1 mmol of active methylene(s) and 1 mmol of corresponding nitroimidazole(s). The reactions were irradiated under 90 °C for 5–10 min. A colored precipitate appeared as the resulting product. The resulting crude solids were filtered and washed with water, followed by methanol. Some of the compounds were purified through column chromatography using DCM: MeOH (9.5:0.5 to 3:1) solvents system over silica. After washing and purification, the solid materials were dried in a vacuum drying oven (Scheme 1). The structures of the compounds are shown in Figure 2.

***3-hydroxy-2-(1-(2-hydroxyethyl)-2-methyl-1H-imidazol-5-yl) cyclohex-2-en-1-one* (1)**
  Yield: 35% (70 mg) as light brown solid; purification: chromatography: DCM: MeOH (9.5:0.5 to 3:1) over silica; m.p = 186–190 °C; FTIR (cm^−1^): V_(C=O)_ = 1684, V_(C=C)_ = 1600, V_(C=N)_ = 1515, V_(C-O)_ = 1276; ^1^H NMR (600 MHz, DMSO-d_6_): δ 14.89 (s, O^12^1H), 7.56 (s, C^8^ 1H), 4.95 (t, J = 5.3 Hz, O ^18^1H), 4.01 (t, J = 5.1 Hz, C^17^2H), 3.64 (t, J = 5.0 Hz, C^16^2H), 2.48 (s, C^19^ 3H), 2.28 (t, J = 6.3 Hz, C^1 & 5^ 4H), 1.82 (p, J = 6.3 Hz, C^6^ 2H); HRMS (ESI^+^): calculated for C_12_H_16_N_2_O_3_ (M + H^+^) 237.27, found 237.12.
***2-(5-(2-hydroxy-6-oxocyclohex-1-en-1-yl)-2-methyl-1H-imidazol-1-yl) ethyl benzoate* (2)**
  Yield: 24% (65 mg) as greenish-yellow solid; purification: filtration, washed with water and methanol; m.p. = 150–154 °C; FTIR (cm^−1^): V_(C=O)_ = 1713, V_(C=C)_ = 1588, V_(C-O)_ = 1266; ^1^H NMR (600 MHz, DMSO-d_6_): δ 7.89 (d, J = 7.7 Hz, C ^23 & 27^2H), 7.63 (t, J = 7.4 Hz, C^25^ 1H), 7.50 (t, J = 7.6 Hz, C^24 & 26^2H), 6.60 (s, C^8^ 1H), 4.33 (t, J = 5.4 Hz, C^17^ 2H), 4.11 (t, J = 5.4 Hz, C^16^ 2H), 2.44 (s, C^19^3H), 2.15 (t, J = 6.3 Hz, C^1 & 5^ 4H), 1.76 (p, J = 6.3 Hz, C^6^ 2H); HRMS (ESI^+^): calculated for C_19_H_20_N_2_O_4_ (M + H^+^) 341.38, found 341.14.
***6-hydroxy-5-(2-methyl-1H-imidazol-5-yl) pyrimidine-2,4(1H,3H)-dione* (3)**
  Yield: total 78% (161 mg) as pink solid; purification: filtration, washed with water and methanol; m.p. = 215 °C decomposed; FTIR (cm^−1^): V_(C=O)_ = 1687, V_(C=O)_ = 1637, V_(C=C)_ = 1580, V_(C-O)_ = 1244; HRMS (ESI+): calculated for C_8_H_8_N_4_O_3_ (M + H^+^) 209.18, found 209.04; ^1^H NMR (600 MHz, DMSO-d_6_): δ 13.15 (s O^12^1H), 9.66 (s, N^1 & 5^ 2H), 7.33 (s, C^8^ 1H), 2.52 (s, C^15^ 3H).
***6-hydroxy-5-(2-methyl-1H-imidazol-5-yl)-2-thioxo-2,3-dihydropyrimidin-4(1H)-one* (4)**
  Yield: total 71% (166 mg) as grey solid; purification: filtration, washed with water and methanol; m.p. = not determined (above 300 °C); FTIR (cm^−1^): V_(C=O)_ = 1644, V_(C=C)_ = 1590, V_(C=N)_ = 1522, V_(C-O)_ = 1272; ^1^H NMR (600 MHz, DMSO-d_6_): δ 13.33 (s, O^12^1H), 13.09 (s, N^16^ 1H), 11.09 (s, N^1 & 5^ 2H), 7.43 (s, C^8^ 1H), 2.53 (s, C^15^ 3H); HRMS (ESI^+^) calculated for C_8_H_8_N_4_O_2_S (M + H^+^) 225.25, found 225.02.
***2-(5-(6-hydroxy-2,4-dioxo-1,2,3,4-tetrahydropyrimidin-5-yl)-2-methyl-1H-imidazol-1-yl) ethyl benzoate* (5)**
  Yield: 69% (249 mg) as white powder; purification: DCM: MeOH (9.5:0.5 to 3:1) over silica; m.p. = 275–280 °C; FTIR(cm^−1^): V_(C=O)_ = 1714, V_(C=C)_ = 1566, V_(C=N)_ = 1515, V_(C-O)_ = 1269; ^1^H NMR (600 MHz, DMSO-d_6_): δ 13.64 (s, O^12^1H), 9.44 (s, N^1 & 5^ 2H), 7.89 (d, J = 7.6 Hz, C^23 & 27^2H), 7.65 (t, J = 7.4 Hz, C^25^ 1H), 7.52 (t, J = 7.6 Hz, C^24 & 26^ 2H), 7.15 (s, C^8^ 1H), 4.45 (t, J = 5.1 Hz, C^17^2H), 4.42 (t, J = 5.2 Hz, C^16^2H), 2.64 (s, C^19^3H); HRMS (ESI^+^): calculated for C_17_H_16_N_4_O_5_ (M + H^+^) 357.34, found 357.11.
***6-hydroxy-1,3-dimethyl-5-(2-methyl-1H-imidazol-5-yl) pyrimidine-2,4(1H,3H)-dione* (6)**
  Yield: total 75% (185 mg) pink solid product; purification: DCM: MeOH (9.5:0.5 to 3:1) over silica; m.p. = not determined (above 300 °C); FTIR (cm^−1^): V_(C=O)_ = 1633, V_(C=C)_ = 1592, V_(C=N)_ = 1555, V_(C-O)_ = 1253; ^1^H NMR (600 MHz, DMSO-d_6_): δ 13.23 (s, O^12^1H), 7.44 (s, C^8^ 1H), 3.31 (s, C^17 & 18^ 6H), 2.53 (s, C^15^ 3H); HRMS (ESI^+^): calculated for C_10_H_12_N_4_O_3_ (M + H^+^) 237.24, found 237.04.
***2-(5-(6-hydroxy-4-oxo-2-thioxo-1,2,3,4-tetrahydropyrimidin-5-yl)-2-methyl-1H-imidazol-1-yl) ethyl benzoate* (7)**
  Yield: 69% (240 mg) dark brown product; purification: filtration, washed with water and methanol; m.p = 225–230 °C; FTIR (cm^−1^): V_(C=O)_ = 1706, V_(C=O)_ 1665 = V_(C=C)_ = 1635, V_(C=N)_ = 1526, V_(C-O)_ = 1275; ^1^H NMR (600 MHz, DMSO-d_6_): δ 13.76 (s, O^12^1H), 10.85 (s, N^1 & 5^2H), 7.89 (d, J = 7.7 Hz, C^23 & 27^2H), 7.64 (d, J = 7.4 Hz, C^25^ 1H), 7.53 (t, J = 7.4 Hz, C^24 & 26^2H), 7.20 (s, C^8^ 1H), 4.44 (t, J = 4.5 Hz, C^17^2H), 4.43 (t, J = 4.6 Hz, C^16^2H), 2.64 (s, C^19^3H). HRMS (ESI^+^): calculated for C_17_H_16_N_4_O_4_S (M + H^+^) 373.41, found 373.09.
***6-hydroxy-5-(1-(2-hydroxyethyl)-2-methyl-1H-imidazol-5-yl) pyrimidine-2,4 (1H, 3H)-dione* (8)**
  Yield: 78% (176 mg) as pale yellow powder; purification: filtration, washed with water and methanol; m.p = not determined (above 300 °C); FTIR (cm^−1^): V_(C=O)_ = 1684, V_(C=C)_ = 1626, V_(C=N)_ = 1547, V_(C-O)_ = 1277; ^1^H NMR (600 MHz, DMSO-d_6_): δ 9.37 (s, N^1 & 5^ 2H), 7.04 (s, C^8^ 1H), 5.05 (s, O ^18^ 1H), 3.95 (t, J = 5.5 Hz, C^17^2H), 3.55 (t, J = 5.4 Hz, C^16^2H), 2.56 (s, C^19^ 3H); HRMS (ESI+): calculated for C_10_H_12_N_4_O_4_ (M + H^+^) 253.23, found 253.09.
***6-hydroxy-5-(1-(2-hydroxyethyl)-2-methyl-1H-imidazol-5-yl)-2-thioxo-2,3-dihydropyrimidin-4(1H)-one* (9)**
  Yield: 76% (193 mg) as pale yellow powder; purification: filtration, washed with water and methanol; m.p. = not determined (above 300 °C); FTIR (cm^−1^): V_(C=O)_ = 1645, V_(C=C)_ = 1584, V_(C=N)_ = 1520, V_(C-O)_ = 1278; ^1^H NMR (600 MHz, DMSO-d_6_): δ 13.07 (s, O^12^1H), 11.10 (s, N^1 &5^2H), 7.54 (s, C^8^ 1H), 5.07 (t, J = 5.5 Hz, O ^18^1H), 4.09 (t, J = 5.1 Hz, C^17^2H), 3.67 (t, J = 4.7 Hz, C^16^2H), 2.58 (s, C^19^3H); HRMS (ESI^+^): calculated for C_10_H_12_N_4_O_3_S (M + H^+^) 269.29, found 269.09.
***6-hydroxy-5-(1-(2-hydroxyethyl)-2-methyl-1H-imidazol-5-yl)-1,3-dimethylpyrimidine-2,4(1H,3H)-dione* (10)**
  Yield: 70% (185 mg) as light pink crystalline solid; purification: filtration, washed with water and methanol; m.p. = 163–165 °C; FTIR (cm^−1^): V_(C=O)_ = 1707, V_(C=C)_ = 1626, V_(C=N)_ = 1562, V_(C-O)_ = 1261; ^1^H NMR (600 MHz, DMSO-d_6_): δ 13.48 (s, O^12^1H), 7.11 (s, C^8^ 1H), 4.98 (t, J = 5.2 Hz, O ^18^1H), 3.93 (t, J = 5.4 Hz, C^17^2H), 3.51 (t, J = 5.3 Hz, C^16^2H), 3.07 (s, C^19 & 20^ 6H), 2.60 (s, C^21^ 3H); HRMS (ESI^+^): calculated for C_12_H_16_N_4_O_4_ (M + H^+^) 281.28, found 281.10.
***2-(5-(6-hydroxy-1,3-dimethyl-2,4-dioxo-1,2,3,4-tetrahydropyrimidin-5-yl)-2-methyl-1H-imidazol-1-yl) ethyl benzoate* (11)**
  Yield: 78% (273 mg) as light pink crystalline solid; filtration, washed with water and methanol; m.p. = 180–185 °C; FTIR (cm^−1^): V_(C=O)_ = 1724, V_(C=O)_ = 1679, V_(C=C)_ = 1575, V_(C-O)_ = 1258; ^1^H NMR (600 MHz, DMSO-d_6_): δ 13.66 (s, O^12^1H), 7.86 (d, J = 7.7 Hz, C^23 & 27^2H), 7.64 (t, J = 7.4 Hz, C^25^ 1H), 7.49 (t, J = 7.6 Hz, C^24 & 26^2H), 7.16 (s, C^8^ 1H), 4.42 (t, J = 4.0 Hz, C^17^2H), 4.41 (t, J = 4.1 Hz, C^16^2H), 3.03 (s, C^28 & 29^ 6H), 2.65 (s, C^19^3H); HRMS (ESI^+^): calculated for C_19_H_20_N_4_O_5_ (M + H^+^) 385.39, found 385.15.

### 2.2. Inhibition of Human CA-II and CA-IX

In vitro inhibition of human CA (CA-II, and CA-IX) was performed in 96-well plate. Each well of the 96-well plate comprised 20 µL of the test compound (0.5 mM), 20 µL of the purified enzyme (0.1 mg/mL), 140 µL of the assay buffer (HEPES–tris, 20 mM), and 20 µL of the substrate (p-Nitrophenyl acetate; prepared in methanol), making a final volume of 200 µL. The assay procedure started with the incubation of the test compound, desired enzyme, and 140 µL of buffer for 15 min. Soon after the incubation, the substrate was added to each well and the plate was placed in the microplate readers (Bio-Rad, Molecular Devices, CA, USA) for 30 min at 25 °C, and the absorbance was recorded at one-minute intervals [28]. All the in vitro testing results, e.g., percent inhibition, and the IC_50_ values were analyzed by EZ-Fit, which is a curve-fitting enzyme kinetics program (Perrella Scientific Inc., Amherst, MA, USA). All kinetic graphs were plotted by GraFit program (Horley, UK). Different parameters and their values, such as the values of intercepts, slopes, correlation coefficients, and their standard error means (SEMs), were calculated by the nonlinear regression analysis using the same program (GraFit program).

### 2.3. In Situ Evaluation of Anticancer Properties 

The anticancer potential of the compounds (**1**–**11**) was determined by MTT (yellow tetrazolium salt, 3-(4, 5-dimethylthizol-2-yl)-2, 5-diphenyl tetrazolium bromide) assay using an aggressive breast cancer cell line, MDA-MB-231. This cell line is ER, PR, and E-cadherin negative, and it lacks the growth factor receptor HER2. It represents a good model of triple-negative breast cancer and is commonly used for in vitro and in vivo studies [31]. The normal human breast cell line (MCF-10A) was used as a control in the study. Cells were cultured in DMEM supplemented with 10% FBS and 1% antibiotics (100 U/mL penicillin). The cells were seeded in a 96-well plate at a density of 1.0 × 10^4^ cells/well and incubated for 24 h at 37 °C with 5% CO_2_. The medium was discarded, and both the cell lines were treated with different concentrations (25 μM, 50 μM, 75 μM, and 100 μM) of synthetic imidazole derivatives [32]. After 48 h of incubation, 20 μL of MTT solution (5 mg/mL) was pipetted into each well and incubated for another 4 h. The medium was later discarded, and the formazan precipitate was dissolved in DMSO. The absorbance of the mixtures was determined by a microplate reader at 570 nm. All the experiments were performed in triplicate, and the cytotoxicity was expressed as a percentage of cell viability and compared with untreated control cells [33].

$$\%\;Viability=\frac{Absorbance\;of\;sample}{Absorbance\;of\;control}\times 100$$

The dose–response and IC_50_ values were calculated by IBM SPSS Statistics 26 software.

### 2.4. Molecular Docking

A well-known bioinformatics method, i.e., docking, was applied to study the binding mechanism of those hybrids with their biological targets. For this purpose, the refined x-ray crystal structures of hCA-IX and hCA-II were retrieved from Protein Data Bank (www.rcsb.org (accessed on 4 June 2022)) with PDB codes of 6RQW [34] (resolution 1.49 Å) and 1BN1 [35] (resolution 2.10 Å), respectively. Using the Molecular Operating Environment software (MOE version 2020.0901) tool [36], the crystal structures were prepared for docking by adding missing hydrogen on residues and partial charges with Amber12: EHT forcefield. Only water molecules within 3Å of the co-crystallized inhibitor were retained in the protein file while all other heteroatoms were deleted. Thereafter, the compounds structures were drawn in ChemBio-Draw and converted into a 3D format by MOE where hydrogen atoms and partial charge (MMFF94x forcefield) were added, and their energy was minimized with an RMSD gradient of 0.1 kcal/mol/Å. For docking, the “Triangle Matcher” placement method of MOE was used, and for scoring, “London dG” scoring function was applied. For each compound, twenty docked poses were saved, and the best docked conformation was selected based on the docking score and maximum binding interactions. The protein–ligand interactions were illustrated by UCSF Chimera [37].

## 3. Results and Discussion

### 3.1. Chemistry

The aromatic nucleophilic substitution reaction is one of the most fundamental chemical reactions, with the replacement of aromatic nitro group(s) by carbon nucleophiles (active methylene(s)).

**Scheme 2:**
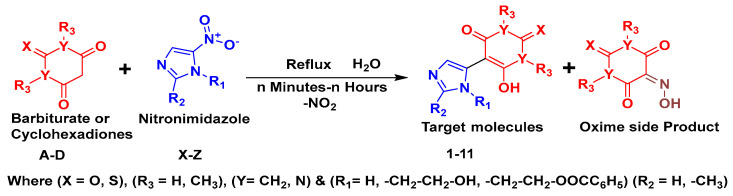
Reflux reaction of active methylenes with nitroimidazoles.

The use of water as an eco-friendly solvent has made the entire process very interesting to synthetic chemists. Chemists often favor organic solvents, yet they are concerning due to their potentially dangerous effects on the environment. Among all solvents, water occupies an undisputed position as the best solvent [37]. In our previous manuscript, we reported a green approach for aromatic nitro group substitution reactions of nitroimidazoles with carbon nucleophiles in water, along with a hypothetical plausible mechanism. Searching for a conceivable reaction route, we tested a variety of solvents (THF, CH3CN, C2H5OH, DCM, and DMSO) for substituting the aromatic nitro group of imidazole with carbon nucleophiles at different pHs (Scheme 2). It was concluded from the multiple experiments that water is the optimum solvent for Scheme 2 [38], while an acidic pH facilitates the removal of the nitro group of imidazole. It was also concluded that carbon nucleophiles were produced in situ as enolized forms in water. These enolized molecules are excellent ambident nucleophiles capable of easily replacing the mono-substituted nitro groups [39].

In our current report, we repeated the same reactions by using the same variety of carbon nucleophiles (active methylenes) and nitroimidazoles as efficient electrophilic reagents. This time, instead of refluxing for 8 min to 80 h, the reaction mixtures were microwave-irradiated to enhance the yields in a short amount of time. As compared to the reflux reactions in Scheme 2, the yields of all target molecules improved by a fair amount in 5–10 min (Scheme 1, entries 1-11). Moreover, just like our previous report, we again observed the formation of an oxime side product by the reaction of carbon nucleophiles (active methylenes) and nitrous acid (HNO_2_) in situ. The oxime yield was 20 to 30% of the total mass of the crude product. The nitrous acid was generated over the course of the reaction. 

### 3.2. In Vitro Testing of Imidazole–Pyrimidine Hybrids against CAs Isozymes

The synthesized imidazole hybrids were tested against human CA-II and human CA-IX (hCA-II and hCA-IX), where these derivatives showed promising inhibitory activities (Table 1). Among the examined hits, compound **7** exhibited strong inhibitory activity for hCA-IX, followed by compounds **3**, **11**, **10**, **4,** and **9**, while compounds **1** and **8** showed comparatively weak activity as compared to the standard “acetazolamide”. Compounds **1**, **2,** and **11** showed potent inhibitory activity for hCA-II, while **3**, **4**, and **9** showed weak activity as compared to acetazolamide. Some of the compounds showed no inhibitory response against any of the isozymes.

### 3.3. Mechanistic Studies

**Figure 3:**
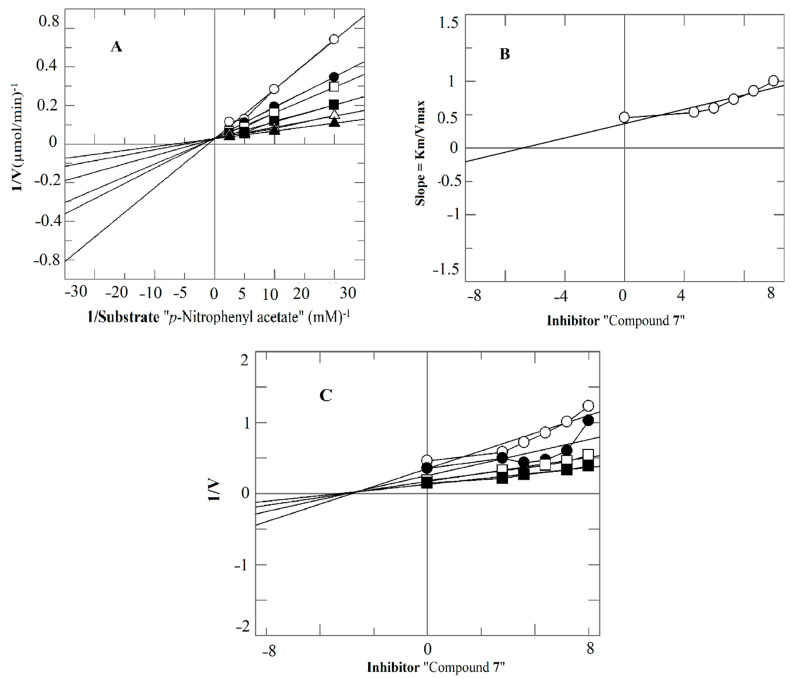
Deducing the type of inhibition of compound **7** against CA-IX. (**A**) The first graph is drawn (Lineweaver–Burk plot) between the reciprocal of reaction rate (velocity) vs. reciprocal of substrate (*p-Nitro phenyl acetate)* in the presence of the highest dose 20 µM (○), second highest dose, and so on: 15 µM (●), 10 µM (**□**), 5 µM (■), 2.5 µM (∆), and negative control (▲) of compound **7**. (**B**) The second graph is (Lineweaver–Burk plot) between the gradient of each line vs. different concentrations of inhibitor “compound **7**”. (**C**) The third graph “Dixon plot” is drawn between reciprocal of reaction rate (velocities) vs. different concentrations of inhibitor “compound **7**”.

**Figure 4:**
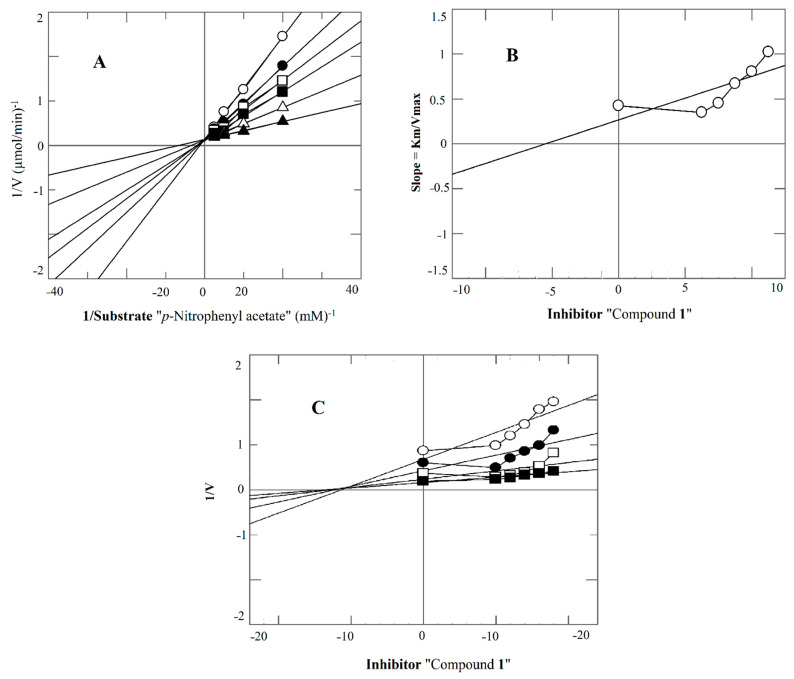
Deducing the type of inhibition of compound **1** against CA-II. (**A**) The first graph is drawn (Lineweaver–Burk plot) between the reciprocal of reaction rate (velocity) vs. reciprocal of substrate (*p-Nitro phenyl acetate*) in the presence of highest dose 25 µM (○), second highest dose, and so on: 20 µM (●), 15 µM (**□**), 10 µM (■), 5 µM (∆), and negative control (▲) of compound **1**. (**B**) The second graph is (Lineweaver–Burk plot) between the gradient of each line vs. different concentrations of inhibitor “compound **1**”. (**C**) The third graph “Dixon plot” is drawn between reciprocal of reaction rate (velocities) vs. different concentrations of inhibitor “compound **1**”.

The in vitro mechanism of action of the potent inhibitors (hCA-IX = **7**, **3**, **11**, **10**, **4** and **9**; hCA-II = **1**, **2** and **11**) was determined by using enzyme kinetic studies in a concentration-dependent manner. Certain parameters of the assay were changed in order to determine the mode of inhibition of compounds against specific enzymes. The compound’s concentration was changed with respect to the IC_50_ values (we used two concentrations above and two concentrations lower than the IC_50_ values). Similarly, four different concentrations of substrate were used (0.25–0.4 mM). Kinetic parameters, such as *Vmax*, *Km*, and *Ki* values of each potent compound were also determined. The kinetic studies indicated that potent inhibitors (**7**, **3**, **11**, **10**, **4**, and **9**) of hCA-IX and (**1**, **2**, and **11**) of hCA-II showed competitive inhibition with a dissociation constant (*Ki*) in the range of 7.32–17.02 µM. These results indicate that all compounds go into active sites of both enzymes. The details of the kinetic parameters are presented in Table 2, and the graphs of mode of inhibition are depicted in Figure 3 and Figure 4.

### 3.4. Specificity of Compounds for Specific Target

When the active compounds were tested against human CA isozymes (hCA-IX and hCA-II), interesting results were obtained. Among them, certain compounds showed specificity against a particular target; for instance, compounds **7**, **10**, and **11** showed specificity towards hCA-IX, while compound **2** showed specificity against hCA-II. These results are noteworthy, as specificity remains a great challenge to address the pathologies associated with CA isozymes. This study highlights particular leads for particular targets. These leads deserve further in vivo mechanistic studies, as they showed excellent results in the in vitro experiments.

### 3.5. Predictive Structure Activity Relationship (SAR) of Imidazole Derivatives in hCA-IX and hCA-II

After the in vitro testing, the compounds with a percentage of inhibition greater than 50 were docked in the crystal structures of their respective enzymes. Our in vitro mechanism-based studies guided us to dock the compounds in the active site of hCA-IX because of their competitive mode of inhibition. The docked conformations of ligands showed that they interact with His200, His140, Trp141, His251, Gln224, Gln203, and Arg196 in the active site of hCA-IX. Additionally, a few water molecules (HOH603, HOH664, and HOH686) in the active site also participated in the protein–ligand bridging.

The most active inhibitor, **7**, mediated three hydrogen bonds (H-bonds) with Asn198 (2.71 Å), Trp141 (2.87Å), and Gln203 (2.44 Å), and bidentate ionic bonds with Arg196 (3.50 Å, 2.71 Å). In addition, two water molecules (HOH664 and HOH552) also provided H-bonds to this molecule. The high potency of **7** in the in vitro and in silico experiments could be due to the presence of ethyl benzoate moiety, which mediated several essential interactions with the active site residues. Similarly, compound **3** mediated two H-bonds with Gln203 at 3.14 Å and Gln224 at 3.02 Å, and three H-bonds with HOH603 (2.00 Å), HOH664 (1.68 Å), and HOH686 (1.36 Å). Compound **4** also mediated three H-bonds and an ionic bond with the catalytic residues and water molecules, including HOH664 (3.87 Å), HOH603 (2.19 Å), His200 (3.45 Å), and Arg196 (2.30 Å), respectively. Other analogs, **10** and **9,** showed slight variation in their inhibitory potency, which was apparent from their binding pattern with the residues. Compound **10** mediated two ionic bonds and a single H-bond with Arg196 with bond lengths of 2.66 Å, 3.94 Å, and 2.66 Å, respectively. Compound **9** mediated H-bonds with Arg196 (2.43 Å), His200 (2.96 Å), and one water molecule, HOH603 (1.74 Å). Both the compounds have an ethyl hydroxy group at same position, with the difference of oxygen and sulfur at “position 2” of ring A (see Scheme 1). Another potent analog, **11**, exhibited lower activity than **7**. The decrease in activity of compound **11** might be due to the presence of two extra methyl and oxygen groups at position 2 in ring A. The docked conformation of **11** showed two H-bonds with His200 (3.50 Å) and HOH66 (3.14 Å), and one hydrophobic bond with Gln203 (2.85 Å). Analogs **1** and **8** showed weak inhibitory activity as compared to the standard. The decrease in activity of **8** might be due to the presence of oxygen at “position 2” of ring A, while in compound **1**, ring A is replaced with cyclohexane instead of pyrimidine. The conformational sampling revealed that compound **1** mediated three H-bonds with His200 (2.90 Å), HOH603 (2.87 Å), and Arg196 (2.61 Å) and an ionic bond with Arg196 (3.78 Å). Similarly, compound **8** formed two H-bonds with His200 (3.16 Å) and Arg196 (2.63 Å), bidentate ionic bonds with Arg196, and one hydrophobic bond with His140.

**Figure 5:**
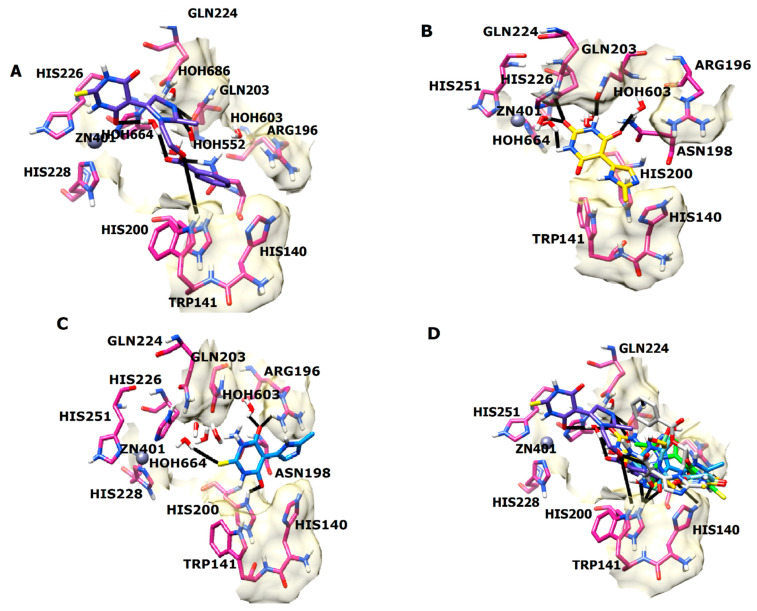
The binding orientation of active compounds is shown in the active site of hCA-IX. (**A**–**C**) The docked pose of the most active compounds, **7**, **3**, and **4**. (**D**) The binding modes of all the inhibitors at the active site of hCA-IX. In the active site, zinc is presented as a slate blue sphere, the ligands are depicted in different colors in stick models, the active site residues are shown in pink stick models, and the hydrogen bonds are presented in black lines.

The SAR clearly demonstrates that the presence of a sulfur group at “position 2” in ring A has a high impact on the potency of the compounds. The compounds with a bulky functional group at ring B and a sulfur group in ring A showed more potent inhibitory activity than the compounds bearing oxygen in ring A, i.e., compounds **3** and **11**. Similar patterns of potency were observed in compounds **8** and **9,** where the sulfur-bearing compound **9** showed potent activity and the oxygen-bearing derivative showed weak activity. In compounds **3** and **4**, this trend of activities changed, which lack bulky groups at ring B; there, oxygen-bearing compounds showed a slightly high potency. The binding interactions of the compounds are tabulated in Table S1 (supporting information) and depicted in Figure 5. 

When these compounds were evaluated against hCA-II, compounds **1**, **2**, and **11** showed potent inhibitory activity, while **3**, **4**, and **9** showed weak inhibitory activities. The active compounds were docked in the crystal structure of hCA-II in order to examine their binding mechanism at the atomic level. The docked pose of compound **1** showed that the compound interacted with the Zn ion in the active site (2.37 Å) and mediated H-bonds with Asn62 (3.12 Å) and His94 (3.01 Å). Compound **2** lost the ionic interaction with the Zn ion but retained H-bonding with Gln92 (3.39 Å), Asn62(2.83 Å), and Thr200 (3.78 Å). Compound **11** was placed near the entrance of the active site cavity, which might be due to the extra methyl group at ring A. However, it mediated three H-bonds with Asn62 (3.09 Å), Glu69 (3.16 Å), and HOH337 (3.34 Å). 

**Figure 6:**
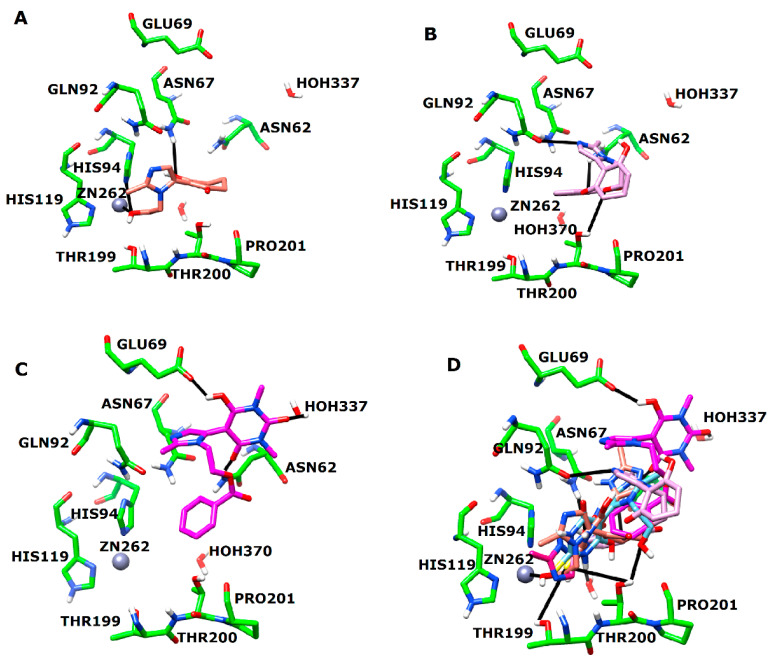
The docked poses of the active compounds in the active site of hCA-II. (**A**–**C**) The binding interactions of most active compounds **1**, **2**, and **4** are shown. (**D**) The binding interactions of all the inhibitors at the active site of hCA-II are shown. In the active site, Zn is presented as a slate blue sphere, ligands are depicted in different colors in stick models, active site residues are presented in green stick models, and H-bonds are presented in black lines.

On the other hand, the weak active inhibitors also showed significant interactions with the residues. Compounds **3** and **4** interacted with Thr199, Asn62, and Asn67. Furthermore, compound **9** formed H-bonds with Pro201, Thr199, and Thr200. The binding interactions of the compounds with hCA-II are presented in Table S1 and Figure 6.

### 3.6. Anticancer Potential of the Synthesized Hybrids

Different concentrations (25 μM, 50 μM, 75 μM, and 100 μM) of imidazole–pyrimidine hybrids (**1–11**) were used to investigate the inhibition of growth of cancer cells in human breast cancer cell line, MDA-MB-231. Simultaneously, the normal human breast epithelial cell line (MCF-10A) was used as a positive control. The MTT [3-(4,5-dimethylthiazol-2-yl)-2,5-diphenyltetrazolium bromide] assay was used to determine the decrease in cancer cell viability induced by the synthesized hybrids. For MDA-MB-231, the IC_50_ values, percent inhibition, and viability of compounds (**1–11**) are given in Table 3. In the MTT assay, all the compounds showed potent activity against MDA-MB-231 cells. Among all the tested hits, compound **5** showed more inhibitory potency in the MDA-MB-231 cell line with an IC_50_ value of 53.65 μM, followed by compounds **8** (55.65 μM), **9** (58.623 μM), **10** (60.93 μM), **2** (62.848 μM), **11** (63.096 μM), **7** (64.009 μM), **4** (75.65 μM), **3** (76.07 μM), **6** (79.43 μM), and **1** (96.01 μM). To determine whether the cytotoxic effects of the compounds were selective for malignant cells as compared to non-malignant cells, the non-tumorigenic (MCF-10A) cells were exposed to the compounds at varying concentrations (25 μM, 50 μM, 75 μM, and 100 μM) in a similar manner as the cancer cells. The results (Table 3) depict that the normal cells are less susceptible to the inhibitory actions of those compounds, particularly to compound **5**, which showed more cell death in breast cancer cells. They also revealed that the triple negative MDA-MB-231 cells, which bear an aggressive phenotype, responded more favorably to most of the compounds with greater cytotoxicity. The diminished cytotoxicity of the compounds in non-tumorigenic cells suggests that these novel compounds will offer promising treatment/therapy for patients with breast cancer.

## 4. Conclusions

Green chemistry is an eco-friendly approach for the synthesis of new hybrids for different beneficial purposes. We report here, for the first time, the synthesis of the imidazole hybrids of pyrimidine through a green chemistry approach. Our synthesized hybrids were then evaluated against human carbonic anhydrase isozymes. In the in vitro analysis, compounds **3**, **4**, **7**, and **9–11** were identified as excellent inhibitors of hCA-IX, while compounds **1**, **2**, and **11** showed good inhibitory activity for hCA-II. The selectivity index showed that compounds **7**, **8**, and **10** are selective inhibitors of hCA-IX, while compound **2** is a selective inhibitor of hCA-II. More importantly, all the active inhibitors are non-cytotoxic for the normal breast cell line, while compound **5** is more toxic to the breast cancer cell line. These newly identified human carbonic anhydrase inhibitors have potential to be considered as therapeutic leads for the treatment of CA-related diseases such as cancer and glaucoma upon further optimization.

## Figures and Tables

**Table 1:** In vitro results of compounds against hCA-II and hCA-IX.

Compounds	hCA-IX	hCA-II
	IC_50_ ± SEM	IC_50_ ± SEM
**1**	23.0 ± 0.5	11.6 ± 0.2
**2**	Inactive	12.3 ± 0.1
**3**	10.9 ± 0.8	30.7 ± 3.4
**4**	17.1 ± 0.6	31.1 ± 1.3
**5**	Inactive	Inactive
**6**	Inactive	Inactive
**7**	9.6 ± 0.2	Inactive
**8**	32.2 ± 1.0	Inactive
**9**	19.9 ± 0.8	27.5 ± 1.3
**10**	14.8 ± 0.1	Inactive
**11**	11.9 ± 0.3	15.3 ± 0.3
**Acetazolamide**	17.21 ± 0.25	18.60 ± 0.26

**Table 2:** Kinetic results of potent active compounds.

Compounds	*Ki* ± SEM	*Vmax	*Vmaxapp	*Km	*Kmapp	Type of Inhibition
**hCA-IX**						
**7**	7.32 ± 0.02	23.01	23.01	0.53	0.41	Competitive
**3**	8.01 ± 0.01	23.01	23.01	0.45	0.38	Competitive
**11**	8.32 ± 0.05	23.01	23.01	0.55	0.42	Competitive
**10**	13.24 ± 0.03	23.01	23.01	0.49	0.40	Competitive
**4**	15.02 ± 0.04	23.01	23.01	0.51	0.43	Competitive
**9**	17.02 ± 0.02	23.01	23.01	0.56	0.47	Competitive
**hCA-II**						
**1**	9.14 ± 0.08	19.32	19.32	0. 28	0.20	Competitive
**2**	11.32 ± 0.02	19.32	19.32	0.26	0.17	Competitive
**11**	12.58 ± 0.04	19.32	19.32	0.24	0.14	Competitive

*Vmax = Maximum velocity of the enzyme in the absence of inhibitors. *Vmaxapp = Maximum velocity of the enzyme in the presence of inhibitors. *Km = Michaelis–Menten constant in the absence of inhibitors. *Kmapp = Michaelis–Menten constant in the presence of inhibitors.

**Table 3:** Percentage of viability and percentage of inhibition of compounds in MDA-MB-231 and MCF-10A cell lines.

Compounds	MDA-MB-231				MCF-10A			
	Concentration (μM)	%Viability	%Inhibition	IC_50_ (μM)	Concentration (μM)	%Viability	%Inhibition	IC_50_ (μM)
**1**	100	48.56	51.44	96.01	100	86.76	13.2	>100
**2**	100	28.43	71.57	62.85	100	84.35	15.7	>100
**3**	100	38.79	61.21	76.07	100	85.65	14.3	>100
**4**	100	39.52	60.48	75.65	100	82.13	17.9	>100
**5**	100	17.93	82.07	53.65	100	79.47	20.5	>100
**6**	100	41.58	58.42	79.43	100	86.81	13.2	>100
**7**	100	30.82	69.18	64.01	100	86.82	13.2	>100
**8**	100	22.25	77.75	55.65	100	84.55	15.5	>100
**9**	100	26.5	73.5	58.62	100	84.5	13.2	>100
**10**	100	24.51	75.49	60.93	100	84.9	15.1	>100
**11**	100	31.82	68.18	63.10	100	87.87	12.1	>100

## Data Availability

Not applicable.

## Supplementary Materials

The following supporting information can be downloaded at: https://www.mdpi.com/article/10.3390/cancers14205079/s1, Table S1. The binding interactions of Compounds in the Active site of hCA-IX and hCA-II.
