# Supplementary material for: Efficient Synthesis with Green Chemistry Approach of Novel Pharmacophores of Imidazole-Based Hybrids for Tumor Treatment: Mechanistic Insights from In Situ to In Silico
Source: Cancers (Basel). 2022 Oct 17;14(20):5079. doi: 10.3390/cancers14205079 (PMC9600394; doi:10.3390/cancers14205079)
Supplement: Supplementary file 1 [file cancers-14-05079-s001.zip › cancers-1942476-supplementary.pdf]

Supporting Information

Efficient Synthesis with Green Chemistry Approach of Novel Pharmacophores of Imidazole-Based Hybrids for Tumor Treatment: Mechanistic Insights from In Situ to In Silico

Majid Khan [1,2,†], Syed Raza Shah [1], Faizullah Khan [1,3,†], Sobia Ahsan Halim [1], Shaikh Mizanoor Rahman [1], Mohammad Khalid [4], Ajmal Khan [1,*], Ahmed Al-Harrasi [1,*]

1 Natural and Medical Sciences Research Center, University of Nizwa, 616 Birkat Al Mauz, Nizwa P.O. Box 33, Oman
2 H.E.J. Research Institute of Chemistry, International Center for Chemical and Biological Sciences, University of Karachi, Karachi 75270, Pakistan
3 Department of Pharmacy, Abdul Wali Khan University Mardan, Mardan 23200, Pakistan
4 Department of Pharmaceutics, College of Pharmacy, King Khalid University, Abha 62529, Saudi Arabia
* Correspondence: ajmalkhan@unizwa.edu.om (A.K.); aharrasi@unizwa.edu.om (A.A.-H.)
† These authors contributed equally to this work.

Table S1. The binding interactions of Compounds in the Active site of hCA-IX and hCA-II.

Compounds	Score	Ligand atom	Receptor atom	Bond type	Bond length (Å)
			**hCA-IX**		
**1**	-3.93	N10	ND1-HIS200	HBA	2.90
		O15	O-HOH603	HBA	2.87
		O17	NH2-ARG196	HBA	2.61
		O17	NH1-ARG196	Ionic	3.78
**3**	-3.49	N5	OE1-GLN203	HBD	3.14
		O14	HE2-GLN224	HBA	3.02
		O14	O-HOH603	HBA	2.00
		N7	O-HOH664	HBA	1.68
			O-HOH686	HBA	1.36
**4**	-3.77	S15	O-HOH664	HBA	3.87
		O14	ND1-HIS200	Ionic	3.45
		N13	NH2-ARG196	HBA	2.30
		O2	O-HOH603	HBD	2.19
**7**	-3.92	O18	NH2-ASN198	HBA	2.71
		O17	NE1-TRP141	HBA	2.87
		N1	OE2-GLN203	HBD	2.44
		O18	O-HOH664	HBD	1.98
		N1	O-HOH552	HBD	2.61
**8**	-3.74	N11	ND1-HIS200	HBA	3.16
		O18	NH2-ARG196	HBA	2.63
		O18	NH1-ARG196	Ionic	3.85
		O18	NH2-ARG196	Ionic	2.63
		6-ring	NE2-HIS140	π-H	2.89
**9**	-3.92	O8	NH2-ARG196	HBA	2.43
		O16	ND1-HIS200	HBA	2.96
		N11	HOH603	HBA	1.74
**10**	-3.93	O18	NH2-ARG196	HBA	2.66
		O18	NH1-ARG196	Ionic	3.94
		O18	NH2-ARG196	Ionic	2.66
**11**	-3.66	O7	ND1-HIS200	HBA	3.50

		O26	O-HOH664	HBA	3.14
		6-ring	NE2-GLN203	π-H	2.85
			**hCA-II**		
**1**	-4.97	O15	ZN-262	Metallic	2.37
		O3	HD2-ASN62	HBA	3.12
		O15	NE2-HIS94	HBA	3.01
**2**	-5.03	N-10	NE2-GLN92	HBA	3.39
		6-ring	ND2-ASN62	π-H	2.83
		O8	HG1-THR200	HBA	3.78
**3**	-4.35	N5	O-HOH370	HBD	3.64
		N10	N-THR199	HBA	3.13
		O15	ND2-ASN62	HBA	2.62
		O15	ND2-ASN67	HBA	2.98
**4**	-4.29	N12	OD1-ASN67	HBD	3.22
		O7	ND2-ASN62	HBA	3.19
		S15	N-THR199	HBA	3.14
		S15	OG1-THR199	HBA	4.02
**9**	-4.58	O16	O-PRO201	HBD	3.06
		S7	N-THR199	HBA	3.74
		O8	OG1-THR200	HBA	2.42

HBA = Hydrogen bond acceptor, HBD = Hydrogen bond donor.
